# Ongoing Transposon-Mediated Genome Reduction in the Luminous Bacterial Symbionts of Deep-Sea Ceratioid Anglerfishes

**DOI:** 10.1128/mBio.01033-18

**Published:** 2018-06-26

**Authors:** Tory A. Hendry, Lindsay L. Freed, Dana Fader, Danté Fenolio, Tracey T. Sutton, Jose V. Lopez

**Affiliations:** a Department of Microbiology, Cornell University, Ithaca, New York, USA; b Halmos College of Natural Sciences and Oceanography, Nova Southeastern University, Dania Beach, Florida, USA; c Department of Conservation and Research, San Antonio Zoo, San Antonio, Texas, USA; University of Texas at Austin

**Keywords:** bioluminescence, evolution, genome reduction, symbiosis, transposons

## Abstract

Diverse marine fish and squid form symbiotic associations with extracellular bioluminescent bacteria. These symbionts are typically free-living bacteria with large genomes, but one known lineage of symbionts has undergone genomic reduction and evolution of host dependence. It is not known why distinct evolutionary trajectories have occurred among different luminous symbionts, and not all known lineages previously had genome sequences available. In order to better understand patterns of evolution across diverse bioluminescent symbionts, we *de novo* sequenced the genomes of bacteria from a poorly studied interaction, the extracellular symbionts from the “lures” of deep-sea ceratioid anglerfishes. Deep-sea anglerfish symbiont genomes are reduced in size by about 50% compared to free-living relatives. They show a striking convergence of genome reduction and loss of metabolic capabilities with a distinct lineage of obligately host-dependent luminous symbionts. These losses include reductions in amino acid synthesis pathways and abilities to utilize diverse sugars. However, the symbiont genomes have retained a number of categories of genes predicted to be useful only outside the host, such as those involved in chemotaxis and motility, suggesting that they may persist in the environment. These genomes contain very high numbers of pseudogenes and show massive expansions of transposable elements, with transposases accounting for 28 and 31% of coding sequences in the symbiont genomes. Transposon expansions appear to have occurred at different times in each symbiont lineage, indicating either independent evolutions of reduction or symbiont replacement. These results suggest ongoing genomic reduction in extracellular luminous symbionts that is facilitated by transposon proliferations.

## IMPORTANCE

Many female deep-sea anglerfishes possess a “lure” containing luminous bacterial symbionts. Here we show that unlike most luminous symbionts, these bacteria are undergoing an evolutionary transition toward small genomes with limited metabolic capabilities. Comparative analyses of the symbiont genomes indicate that this transition is ongoing and facilitated by transposon expansions. This transition may have occurred independently in different symbiont lineages, although it is unclear why. Genomic reduction is common in bacteria that only live within host cells but less common in bacteria that, like anglerfish symbionts, live outside host cells. Since multiple evolutions of genomic reduction have occurred convergently in luminous bacteria, they make a useful system with which to understand patterns of genome evolution in extracellular symbionts. This work demonstrates that ecological factors other than an intracellular lifestyle can lead to dramatic gene loss and evolutionary changes and that transposon expansions may play important roles in this process.

## INTRODUCTION

**FIG 1:**
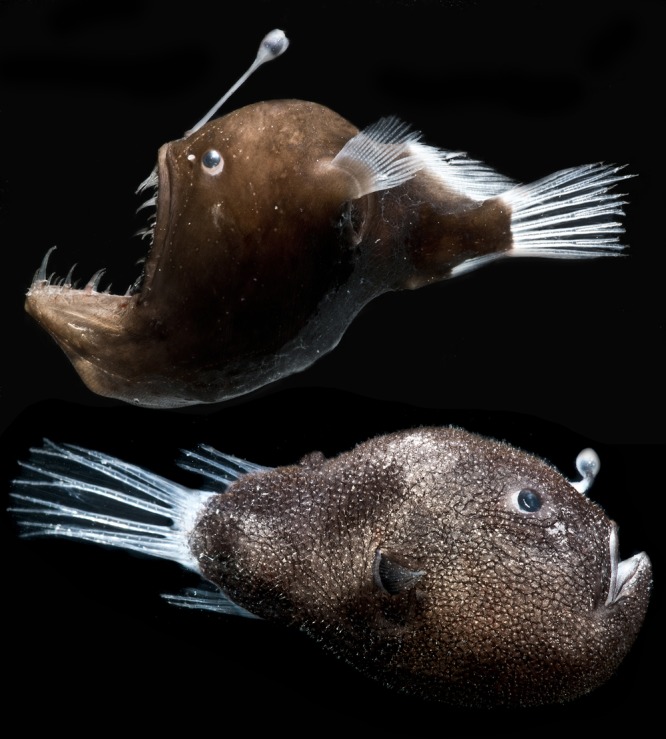
Female deep-sea anglerfish. Shown are adult female specimens of M. johnsonii (top) and C. couesii collected on DEEPEND Consortium cruises in similar locations to those of our samples. (Photo credit: Danté Fenolio, San Antonio Zoo.)

Bioluminescent symbiosis between bacteria and marine fishes and squid has evolved independently many times, with 2 origins in squid (1) and at least 17 origins across fishes, for a total of over 460 bacterially luminous host species (2, 3). Repeated evolutions of bioluminescent symbiosis are likely due to the benefits derived by hosts, which use bacterially produced light to avoid predators and find prey and for intraspecific signaling (4). Hosts maintain luminous bacteria in specialized structures called light organs and provide them with nutrients (5–7). Diverse host species include coral-reef-dwelling, coastal, and pelagic fishes, as well as poorly studied species from the deep sea, such as deep-sea anglerfishes of the suborder Ceratioidei (Teleostei: Lophiiformes). On a global scale, Ceratioidei is notable in that it is the most speciose fish taxon in the bathypelagic zone (oceanic waters deeper than 1,000 m), which is by far Earth’s largest ecosystem (8, 9). A common feature of the taxon is that females of the majority of species have light organs in the form of lures, or escae, extending from the illicium, a modified dorsal fin ray on their heads. These light organs contain dense, extracellular populations of bacteria and emit light through transparent tissue (Fig. 1) (8, 10). However, ceratioids remain the least-characterized group of bacterially luminous fishes, and little is known about their symbionts.

The majority of bacterially luminous fish species engage in symbiosis with just six species of bacteria in the genera *Aliivibrio* and *Photobacterium* from the *Gammaproteobacteria* family *Vibrionaceae* (2, 11). In contrast, ceratioids associate with luminous bacteria that are related to other *Vibrionaceae* species, but may be distinct lineages (12). *Aliivibrio* and *Photobacterium* symbiont species are all facultatively symbiotic and form free-living populations in multiple habitats in addition to engaging in symbiosis with hosts (2). In keeping with this habitat diversity, members of *Vibrionaceae* have relatively large (~5 Mb) and diverse genomes (13). Although bioluminescent symbiosis appears to have evolved several times in different *Vibrionaceae* lineages (11), most luminous symbionts show only small-scale genomic changes in response to host interactions (14–16).

One exception to this pattern are the luminous symbionts in the genus “*Candidatus* Photodesmus,” which are obligately dependent on their hosts, flashlight fishes (Anomalopidae), for growth (7, 17). These bacteria are extracellular (18, 19) and appear to colonize new hosts from the environment rather than directly from host-associated populations (5, 17). However, their genomes are reduced by 80% compared to those of free-living relatives, and their loss of metabolic genes indicates that they are unable to establish free-living populations in most environments (7, 17). “*Ca.* Photodesmus” genomes also show very similar molecular patterns to intracellular, vertically transmitted symbionts, including high substitution rates and relaxed purifying selection (20, 21).

Intracellular bacterial symbionts, including the mutualists of many insects, show a striking pattern of convergently derived genome reduction (22–25). Physical restriction to host cells, sharing metabolic products, and vertical transmission between host generations are thought to lead to extreme gene loss in multiple ways. First, restriction to a host cell relaxes selection on functions only needed outside the host, allowing for the loss of genes underlying those functions. Second, host-restricted bacteria experience reductions in effective population sizes (*N*_*e*_) due to a lack of opportunities for recombination and horizontal gene transfer, as well as population bottlenecks during transmission between host generations. This decrease in *N*_*e*_ increases the effect of genetic drift and the rate of nucleotide substitutions and gene loss (26–28). Together these processes are thought to lead to relatively fast genomic degeneration immediately following host restriction and continued reduction to minimal genomes (24, 29).

Recently derived intracellular symbionts sometimes show remnants of this ongoing genomic degeneration, such as high numbers of pseudogenes or transposable elements (TEs) (29–33). This proliferation of TEs is thought to be caused by relaxed selection on TE regulation, as well as relaxed purifying selection on genes throughout the genome that serve as potential insertion sites. TEs may facilitate the process of genomic reduction by inserting within and disrupting genes (30, 31). These genomic elements are eventually lost from genomes by deletion, as they are not typically seen in relatively long-term intracellular symbionts (24, 34). They also do not tend to accumulate in free-living bacteria, presumably because of selection against insertion into required genes (35).

Extracellular symbionts, particularly those that are horizontally transmitted or acquired from the environment, are not physically restricted to hosts and therefore do not typically undergo gene loss (25, 36, 37), with a few known exceptions (7, 38, 39). Like all known luminous bacteria, ceratioid symbionts are extracellular, and pores present on the surface of light organs may allow movement of the bacteria between light organs and the environment (19). However, previous work has stated that anglerfish symbionts are unculturable, suggesting the potential for obligate dependence on hosts and possibly genomic patterns of evolution similar to those of flashlight fish symbionts (12). To resolve the evolutionary histories of deep-sea anglerfish symbionts, we generated and analyzed *de novo* genome sequences for the light organ symbionts from two distantly related ceratioid host species, two individuals of Cryptopsaras couesii (Ceratiidae) and one individual Melanocetus johnsonii (Melanocetidae).

## RESULTS AND DISCUSSION

### Genetic diversity, evolutionary relationships, and rates.

Symbiont genome sequences from the two ceratioid fish species show a high degree of host specificity and little genetic diversity. The symbiont genomes from separate C. couseii specimens (CC26 and CC32) were extremely similar, sharing 99.9% nucleotide sequence identity across entire genome alignments. Furthermore, previously reported 16S rRNA gene sequences from C. couseii and M. johnsonii specimens caught in 1990 near the Canary Islands (12, 40) were each 100% identical across 1,416 bp to those sequences from the C. couseii and M. johnsonii (MJ02) symbionts sequenced here. We also observed very little intralight organ diversity within each sample. The majority (>90%) of possible alternate bases found in the reads from the CC32 and MJ02 libraries were present in less than 0.05% of the read depth at each site, suggesting that they are sequencing errors (see Fig. S1 in the supplemental material). Additionally, alternate bases present in greater than 1% of the read depth, which might represent actual intralight organ variation, were found at a rate of only 1.6 potential polymorphisms per kilobase. Although these samples sizes are low and this pattern could change with further sampling, the low level of genetic diversity found within each anglerfish symbiont species and the apparent symbiont specificity to a host species are unusual compared to those of free-living luminous symbionts of fishes (11, 21) and very similar to those of previously reported patterns in flashlight fish symbionts (21) and obligate symbionts (41, 42). This pattern could result from small, possibly monoclonal founding populations of bacterial cells within the esca and/or low genetic diversity across symbiont populations.

10.1128/mBio.01033-18.2 FIG S1  Frequency spectrum of alternate bases present in symbiont genome reads from the CC32 library (A) and MJ02 library (B). The majority of alternate bases in each library are at very low frequency (<0.05%), suggesting that they are errors rather than intralight organ variation of the symbiont population. Download FIG S1, DOCX file, 1 MB. Copyright © 2018 Hendry et al. 2018 Hendry et al. This content is distributed under the terms of the Creative Commons Attribution 4.0 International license.

**FIG 2:**
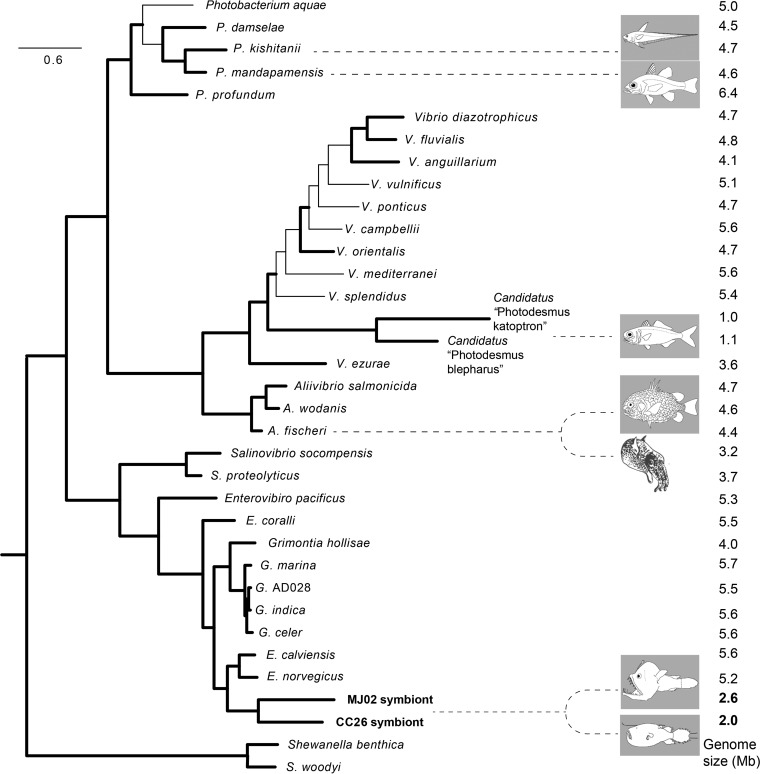
Relationship of anglerfish symbionts to major relative clades. Shown is a maximum likelihood phylogenomic tree based on 253 conserved protein sequences obtained from PhyloPhlAn. Nodes with bootstrap support values over 95% are shown with bold branches. Representative fish and squid host pictures are shown to indicate luminous symbionts. Genome size values are indicated in the far right column and were taken from GenBank. The anglerfish symbiont sequences from CC26 and MJ02 are shown in bold.

Phylogenetic analyses recovered anglerfish symbiont genotypes from C. couesii and M. johnsonii as sisters to each other with high support (Fig. 2; see Fig. S2 in the supplemental material). The symbionts were placed within a clade containing members of the genus *Enterovibrio*, closely related to Enterovibrio calviensis and Enterovibrio norvegicus. The relatively short branch separating the anglerfish symbiont clade from *Enterovibrio* strains, as well as their position nested within the genus, supports the placement of the symbionts within the genus *Enterovibrio*. However, the long branches separating the symbionts of different fish species, as well as their low average nucleotide identity (ANI), which was only 72.4% among shared coding loci, supports the separation of the symbionts from each fish species into separate bacterial species. We propose the names “*Candidatus* Enterovibrio luxaltus” (“deep light”) and “*Candidatus* Enterovibrio escacola” (“esca [bait] dwelling”) for the CC26/CC32 and MJ02 symbionts from C. couesii and M. johnsonii, respectively.

10.1128/mBio.01033-18.3 FIG S2  Maximum likelihood phylogenetic tree using 7 housekeeping genes (the 16S rRNA gene, *atpA*, *gapA*, *gyrB*, *pyrH*, *rpoA*, and *topA*). Analysis was done in IQTree using a general time reversible model (chosen by IQTree) and 1,000 bootstrap replicates. Bootstrap values of >80% are shown below nodes. Download FIG S2, DOCX file, 0.4 MB. Copyright © 2018 Hendry et al. 2018 Hendry et al. This content is distributed under the terms of the Creative Commons Attribution 4.0 International license.

The “*Ca.* Enterovibrio escacola” and “*Ca.* Enterovibrio luxaltus” lineages are each separated from other taxa by long branches, suggesting that they may be evolving at an elevated rate compared to relatives. To test this, we compared the likelihoods of molecular clock models that assumed distinct relative substitution rates for anglerfish symbionts and relatives. Allowing anglerfish symbionts to evolve at a distinct rate led to a significant increase in likelihood over the null hypothesis of a global clock (Table 1). The same result was found in this analysis for flashlight fish symbionts, which have previously been shown to be evolving more quickly than free-living relatives (20), and was true for both nucleotide and amino acid sequence data. This was not the case for clades containing free-living relatives, either facultative symbionts or nonsymbiotic species, which were generally evolving more slowly than the rest of the tree (Table 1). The anglerfish symbiont clade was estimated to be evolving at 3.5 times the rate of relatives at the nucleotide level and 4.8 times the rate of relatives in protein sequence, higher rates than the estimates for flashlight fish symbiont evolution compared to relatives. These results confirm that the anglerfish symbionts are evolving at an increased rate compared to free-living relatives and have accumulated a higher relative number of nonsynonymous substitutions.

**TABLE 1:** Comparison of molecular clock models and relative substitution rates using either 7 housekeeping gene sequences or 253 conserved protein sequences^a^

Tree	df	Clade(s) with distinct rate(s)	LR	Null (−lnL0)	Alternative (−lnLA)	*P^b^*	Relative substitution rate
Housekeeping	1	Anglerfish symbionts	269.87	33,576.04	33,441.11	0.0000*	3.5
	1	Flashlight fish symbionts	264.18	33,576.04	33,443.95	0.0000*	2.5
Protein	1	Anglerfish symbionts	488.81	57,332.26	57,087.86	0.0000*	4.8
	1	Flashlight fish symbionts	340.63	57,332.26	57,161.95	0.0000*	2.4
Housekeeping	1	E. calviensis and E. norvegicus	12.26	33,576.04	−33,569.91	0.0005*	0.7
Protein	1	E. calviensis and E. norvegicus	55.59	57,332.26	−57,304.47	0.0000*	0.6
Housekeeping	1	*Grimontia*	0	33,576.04	−33,576.04	1.0000	0.6
Protein	1	*Grimontia*	8.54	57,332.26	−57,327.99	0.0040*	0.7
Housekeeping	1	*Aliivibrio*	0	33,576.04	−33,576.04	1.0000	0.6
Protein	1	*Aliivibrio*	80.44	57,332.26	−57,292.05	0.0000*	0.6
Housekeeping	1	*Photobacterium*	126.52	33,576.04	−33,513.78	0.0000*	0.5
Protein	1	*Photobacterium*	96.04	57,332.26	−57,284.25	0.0000*	0.6

a Abbreviations: df, degrees of freedom; LR, likelihood ratio; −lnL0, log likelihood null model; −lnLA, log likelihood alternative model.

b *, significant likelihood ratio test result.

### Genome assemblies.

The genome from the C. couesii CC26 sample assembled into two plasmids recovered as circular (CC26 P1 and P2) and two large contigs possibly matching to chromosomes I and II of *Vibrionaceae* taxa (Table 2). The CC32 assembly was highly similar (discussed above) but contained more contigs, so we will present data for just the better-assembled CC26 genome hereafter. The M. johnsonii MJ02 symbiont assembly contained 39 contigs, including four circular plasmids. Based on assembly coverage depth, all plasmids had a similarly low copy number (1 to 3 copies per genome). All three symbiont assemblies were found to contain conserved protein coding genes typically used to assess genome completeness (43, 44), indicating that they are nearly fully complete in sequence. The “*Ca.* Enterovibrio luxaltus” genome assembled at 2.1 Mb, and the “*Ca.* Enterovibrio escacola” genome totaled 2.6 Mb. Both genomes had a high number of predicted pseudogenes (785 and 974, respectively), a low number of rRNA genes (1 operon plus an additional 5S rRNA gene in “*Ca.* Enterovibrio luxaltus” and 1 operon in “*Ca.* Enterovibrio escacola”), and a low number of tRNA genes (Table 2).

**TABLE 2:** Overview of genomic features of the symbionts of three anglerfish specimens (two C. couesii and one M. johnsonii) compared to the genomes of symbionts from two flashlight fish (Anomalops katoptron and Photoblepharon palpebratus), two free-living, nonsymbiotic relatives, and a facultative symbiont of Euprymna scolopes^a^

Host	Bacterium	Specimen or strain	Genome size (bp)	Contigs/plasmids	Ecology	Coverage	*N*_50_ (reference)	G+C %	Total CDS	Complete CDS	Pseudogenes	rRNA/tRNA
C. couesii	E. luxaltus	CC26	2,143,356	4/2	Obligate symbiont	14x	1,604,691 (this study)	37.7	2,447	1,662	785	4/36
C. couesii	E. luxaltus	CC32	2,013,547	11/1	Obligate symbiont	81x	249,450 (this study)	37.6	NA	NA	NA	NA
M. johnsonii	E. escacola	MJ02	2,645,619	39/4	Obligate symbiont	58x	113,883 (this study)	39.8	3,290	2,316	974	3/39
A. katoptron	P. katoptron	Akat8	1,015,921	NA/1	Obligate symbiont	NA	NA (17)	30.8	933	873	13	15/32
P. palpebratus	P. blepharus	Ppalp1	1,112,309	NA/2	Obligate symbiont	NA	NA (17)	35.6	1,003	932	23	15/33
NA	V. campbellii	ATCC BAA-1116	6,058,377	NA/1	Free-living	NA	NA (75)	45.4	5,798	5,210	435	32/121
NA	E. norvegicus	FF-33	5,160,129	NA/unknown	Free-living	NA	NA (76)	47.4	4,664	4,929	188	8/69
E. scolopes	A. fischeri	ES114	4,272,718	NA/1	Facultative symbiont	NA	NA (52)	38.3	3,814	3,654	5	37/118

a CDSs, coding DNA sequences; NA, not applicable.

Consistent with their role as luminous symbionts, both genomes contain luminescence genes *luxCDABEG* in the same operon structure seen in other luminous bacteria (2). We were unable to determine if these genes may be regulated by quorum sensing, as is true in some other luminous species such as Aliivibrio fischeri and Vibrio harveyi, because we found no orthologs of known luminescence regulatory genes in the symbiont genomes (45, 46). No luminous strains have previously been reported in the early branching *Vibrionaceae* genera *Enterovibrio*, *Grimontia*, or *Salinivibrio*. Phylogenetic analysis of the anglerfish symbiont *lux* genes does not show high relatedness with *lux* genes from other taxa, which could suggest horizontal gene transfer (see Fig. S3 in the supplemental material), although we cannot exclude this possibility. The inclusion of luminous anglerfish symbionts within the genus *Enterovibrio* suggests a possible earlier evolution of luminescence than previously thought and supports the hypothesis that luminescence arose ancestrally in *Vibrionaceae* and has been lost in many lineages (47).

10.1128/mBio.01033-18.4 FIG S3  Maximum likelihood phylogenomic tree compared to a maximum likelihood phylogeny of *lux* luminescence genes. Phylogenomic analysis was done using PhyloPhlAn with the strains in Table S1, and *lux* gene analysis was done in IQTree using a general time reversible model (chosen by IQTree) and 1,000 bootstrap replicates. Branches with >80% bootstrap support are shown in bold. Download FIG S3, DOCX file, 0.4 MB. Copyright © 2018 Hendry et al. 2018 Hendry et al. This content is distributed under the terms of the Creative Commons Attribution 4.0 International license.

10.1128/mBio.01033-18.10 TABLE S1  Strains and accession numbers of sequences used in this study. Download TABLE S1, XLSX file, 0.1 MB. Copyright © 2018 Hendry et al. 2018 Hendry et al. This content is distributed under the terms of the Creative Commons Attribution 4.0 International license.

### Genome reduction.

The total genome size for both symbiont species (2 and 2.6 Mb) is reduced by about 50% compared to the genomes of free-living relatives (Fig. 2). The closest relatives to the symbionts, free-living *Enterovibrio* strains, have 5- to 5.5-Mb genomes, and the average across free-living *Vibrionaceae* species is about 5 Mb (values from GenBank). This reduction is even more extreme when considering just functional protein coding genes (non-pseudogenes). The three most closely related *Enterovibrio* strains with sequenced genomes average about 5,200 predicted functional protein coding genes, whereas the anglerfish symbionts have 1,662 (CC26) and 2,316 (MJ02), 68% and 55% reductions in predicted functional coding sequence number. The phylogenetic reconstruction (Fig. 2) shows that the genomic reductions in anglerfish symbionts and flashlight fish symbionts represent two convergent evolutions among luminous symbionts. All other known groups of luminous symbionts are free-living and have larger (~4.5 Mb) genomes. Genome reduction can be caused by relaxed purifying selection at a genome-wide scale, as is found in bacteria that have become obligately associated with hosts (24, 48). Similar to obligate symbionts, the anglerfish symbiont genomes are reduced, evolving at an elevated evolutionary rate compared to relatives, and contain a large number of pseudogenes. These changes are consistent with genome-wide relaxed selection and high genetic drift in the anglerfish symbionts and suggest that they may be undergoing genome reduction due to the host association, rather than due to genome streamlining, as can be found in some marine bacteria (49, 50).

### Gene content and inferred ecology.

**FIG 3:**
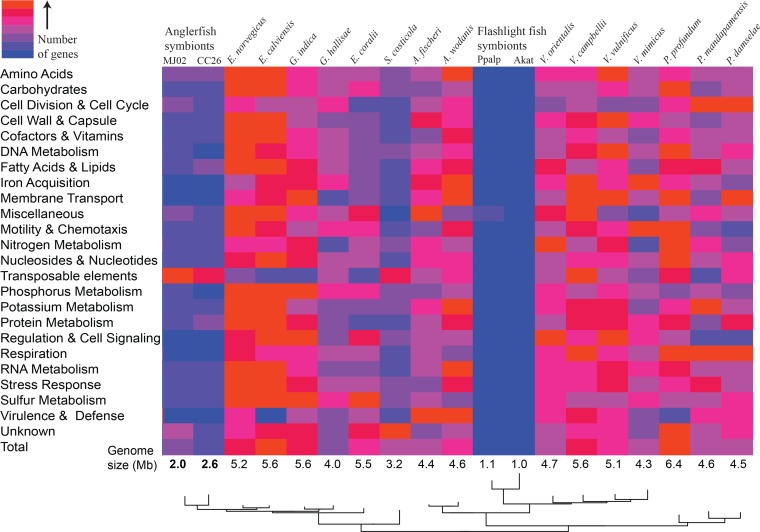
Numbers of genes by functional category. Shown is a heat map of gene content in the genomes of anglerfish symbionts, flashlight fish symbionts, and free-living *Vibrionaceae* members (all other columns). Columns are ordered by relatedness based on a phylogeny (adapted from Fig. 2). Colors were assigned based on the distribution of genes within each functional category. Genomes were taken from GenBank, and categories are based on RAST classifications. For specific numbers of genes, see the table version of this figure (Fig. S5).

The overall gene content of anglerfish symbiont genomes (Fig. 3) is dramatically different from most free-living *Vibrionaceae* species, including closely related *Enterovibrio* species. One possible exception to this pattern is the genomes of *Salinivibrio* species (Fig. 3). This genus is typically isolated from hypersaline environments such as salt lakes or salted meats (51). Because these strains are the free-living *Vibrionaceae* taxa with the smallest known genomes, and because they share some potential similarities with anglerfish symbionts, such as gene loss and increases in transposable element numbers (discussed below), we performed some focused comparisons between Salinivibrio costicola subsp. costicola and anglerfish symbionts. Although the anglerfish symbiont genomes look somewhat similar to S. costicola subsp. costicola at a broad scale, there are many notable differences at a finer scale (Table 3). *Salinivibrio* species have similar numbers of metabolic genes, including phosphotransferase system (PTS) genes for sugar uptake, and genes involved in amino acid synthesis and energy metabolism, compared to other free-living relatives. Anglerfish symbiont genomes have only one PTS, which is specific to glucose, and have a marked reduction in complete amino acid synthesis pathways (see Fig. S4 in the supplemental material) and total numbers of energy metabolism genes. The anglerfish symbiont genomes have also lost most of the DNA repair and recombination genes typically found in relatives, a pattern that is widely shared among obligate and intracellular symbiotic bacteria (52).

10.1128/mBio.01033-18.5 FIG S4  Loss of amino acid synthesis pathways in anglerfish symbionts. Amino acid synthesis pathways present in relatives (taken from KEGG pathways from Vibrio campbellii ATCC BAA-1116) are shown, with gene presence or absence in anglerfish symbiont genomes color coded. Complete pathways are indicated by colored amino acid products. Numbered circles represent enzymatic steps in the pathway, and their corresponding enzymes are listed on the right. Download FIG S4, DOCX file, 0.3 MB. Copyright © 2018 Hendry et al. 2018 Hendry et al. This content is distributed under the terms of the Creative Commons Attribution 4.0 International license.

10.1128/mBio.01033-18.6 FIG S5  Numbers of genes in functional categories for free-living *Vibrionaceae* members, anglerfish symbionts, and flashlight fish symbionts. Genes may be present in multiple categories. Download FIG S5, DOCX file, 1.4 MB. Copyright © 2018 Hendry et al. 2018 Hendry et al. This content is distributed under the terms of the Creative Commons Attribution 4.0 International license.

**TABLE 3:** Numbers of genes in highly reduced or retained categories in the anglerfish symbionts (CC26 and MJ02 samples), flashlight fish symbiont (Anomalops katoptron symbiont Akat8), and free-living relatives (E. calviensis, S. costicola subsp. costicola, and A. fischeri)

Category	No. of genes in category for:					
	Free-living relative			Symbiont		
	*Enterovibrio*	*Salinivibrio*	*Aliivibrio*	Flashlight fish	CC26	MJ02
Reduced categories						
PTS (type)	12 (varied)	11 (varied)	13 (varied)	1 (glucose)	1 (glucose)	1 (glucose)
Amino acid synthesis (complete pathways)	91 (20)	74 (19)	85 (20)	18 (4)	26 (7)	34 (9)
Energy metabolism	661	202	312	49	83	84
DNA repair and recombination	12	11	12	12	2	3
Methyl-accepting chemotaxis proteins	36	20	43	2	1	3
Retained categories						
Cell wall synthesis	253	120	232	76	103	116
Motility and chemotaxis	150	102	131	57	89	89

Due to the small genome size of anglerfish luminous symbionts compared to close relatives, we assume that genes inferred to be functional within the genomes have been retained because they are necessary for growth or are ecologically beneficial, whereas genes common in relatives but lacking in the symbiont genomes have been lost due to decreased selection. This pattern of gene retention allows for some inference about the likely ecological lifestyle of symbionts, although we cannot rule out the possibility that any individual gene may have been retained by chance. For instance, the limited metabolic capabilities of the anglerfish symbionts suggest that they must acquire glucose and many amino acids from the environment and that anglerfishes must be supplying these nutrients to their symbionts (7). These metabolic limitations further suggest some degree of host dependence or restriction to marine environments where glucose and amino acids would be regularly available, which may be scarce in the deep sea. Patterns in other functional categories that are typically underrepresented in bacteria adapted to stable environmental conditions also support this hypothesis (53). Genes in the categories of membrane transport, regulation and cell signaling, and stress responses are all highly reduced in anglerfish symbionts compared to free-living relatives (see Fig. S5 in the supplemental material). Members of the *Vibrionaceae* are typically metabolically diverse; they are found in many marine habitats and associate with many types of hosts (13, 54). The evolutionary switch to a more limited environmental range and possible dependence on hosts for growth appears to be rare among host-associated or bioluminescently symbiotic *Vibrionaceae* species, having only been previously described in the luminous symbionts of flashlight fish, which also rely on hosts for glucose and amino acids (7).

The genomic evidence suggests that despite some host dependence, like all other known luminous symbionts, these bacteria may leave the light organ and persist outside the host. Although all broad functional categories show some reduction in gene number, some pathways that are typically lost in obligate or intracellular symbionts are retained in the anglerfish symbionts. Notably, a relatively large number of genes involved in cell wall synthesis are found in the anglerfish symbiont genomes, suggesting that they can synthesize a robust cell wall. These are typically lost in host-dependent symbionts, even those that are extracellular (38). Furthermore, the bacteria have retained genes suggesting that they are chemotactic and motile. This includes all nearly 60 genes necessary for production of flagella and transmission of chemotaxis signals. In contrast, many accessory genes, which are not required for these functions, have been lost (Table 3). Although components of these pathways are sometimes retained, full pathways for chemotaxis and motility are universally lost in known obligate symbionts (17, 55–58). Electron micrographs from inside anglerfish light organs show densely packed bacterial populations where motility and chemotaxis are unlikely to be useful (10). Therefore, these functions may be used primarily outside the host. In light of the large-scale loss of genetic pathways across the symbiont genomes, the apparent selection to retain pathways useful mainly outside the host suggests that an extrahost phase may be an important part of the symbiont’s lifestyle. This is also seen in flashlight fish symbionts, which have retained genes in the categories of cell wall synthesis and motility and are known to persist and be motile in seawater outside the host (17).

Although the anglerfish symbionts have intact chemotaxis and motility pathways, they show a large reduction in genes coding for methyl-accepting chemotaxis proteins (MCPs), cell surface proteins that detect chemical signals in the environment and elicit a motility response (59, 60). Whereas most *Vibrionaceae* species, including *Salinivibrio* and *Enterovibrio* species, have 20 to 50 MCP genes specific to varied ligands, the “*Ca.* Enterovibrio luxaltus” and “*Ca.* Enterovibrio escacola” genomes contain only one or three functional MCP genes, respectively (Table 3). The predicted function of the anglerfish symbiont MCP genes is not apparent by comparison to genes of known function. Two of the MCP genes found in the MJ02 genome, as well as the one CC26 MCP gene, cluster very closely in phylogenetic analysis and are related to an MCP gene with unknown function retained in a flashlight fish symbiont genome (see Fig. S6 in the supplemental material). The additional MJ02 MCP gene is more distantly related. The anglerfish symbionts appear to have relatively restricted chemotaxis abilities, presumably detecting only a small number of attractants or repellants in the environment. This may indicate that the bacteria are not actively searching for nutrient-rich habitats but may focus on chemicals associated with their specific habitat, such as chemical cues from hosts.

10.1128/mBio.01033-18.7 FIG S6  Maximum likelihood phylogenetic tree of methyl-accepting chemotaxis protein (MCP) sequences from anglerfish symbionts, flashlight fish symbionts, and free-living members of *Vibrionaceae*. Analysis was done in IQTree using a general matrix of amino acid exchange rates, empirically determined amino acid frequencies, a gamma distribution with four categories for rate heterogeneity, and 1,000 bootstrap replicates. Branches are color coded by bootstrap value. MCP genes previously demonstrated to contain conserved amino acid ligand binding domains (T. A. Hendry, J. R. de Wet, K. E. Dougan, and P. V. Dunlap, Genome Biol Evol 8:2203–2213, 2016, https://doi.org/10.1093/gbe/evw161) are highlighted. Sequences were taken from GenBank, with the addition of anglerfish symbiont MPC proteins from this study. Download FIG S6, DOCX file, 0.7 MB. Copyright © 2018 Hendry et al. 2018 Hendry et al. This content is distributed under the terms of the Creative Commons Attribution 4.0 International license.

The anglerfish symbionts have also retained genes for carbon storage and utilization in the form of polyhydroxybutyrate (PHB). Host-associated bacteria typically lose pathways involved in carbon storage (61), but flashlight fish symbionts have retained multiple types of carbon storage pathways (*phbCAB* in both species, glycogen storage in one), which have been hypothesized to be useful for their known persistence outside the host (17). Both flashlight fish symbionts and anglerfish symbionts from an M. johnsonii specimen show occlusions in electron micrographs of host light organs that appear to be PHB granules, supporting the expression of the genes for carbon storage within the host environment (10, 18). Under this model, the fish host supplies an excess of carbon (in the form of glucose), as well as other nutrients to the bacteria in the light organ. The symbionts store excess carbon as PHB granules, which can later be used as a carbon and energy source outside the host. However, the metabolic limitations of the anglerfish symbionts mean that after release from the light organ, finding another habitat that can supply glucose and amino acids, such as another fish host, is imperative. This model then also explains the retention of pathways for chemotaxis and motility, which may be useful in finding new hosts or habitats.

### Transposable element expansion.

Both the “*Ca.* Enterovibrio luxaltus” and “*Ca.* Enterovibrio escacola” genomes contain extremely high numbers of transposable elements (TEs), specifically transposons (Table 4). The “*Ca.* Enterovibrio luxaltus” genome contains 691 TE genes, all but two of which are transposases, and the “*Ca.* Enterovibrio escacola” genome contains 921 TE genes, most of which are transposases. These genes account for 28% and 31%, respectively, of the total coding sequences in the genomes, the highest percentage per bacterial genome that we found previously reported (29–33, 35, 62). In comparison, free-living *Vibrionaceae* species, like other free-living bacteria, have relatively few TE genes. Here we focused on Salinivibrio costicola subsp. costicola, which is closely related to the genus *Enterovibrio* and has the largest number of TE genes of the genomes analyzed here (excluding anglerfish symbionts), yet those genes account for only 2% of the coding sequence in the genome (Table 4).

**TABLE 4:** Transposable element features of the anglerfish symbionts (CC26 and MJ02 samples), flashlight fish symbiont (A. katoptron symbiont Akat8), and S. costicola subsp. costicola genomes

Parameter	Result for:			
	Symbiont			*Salinivibrio* genome
	CC26	MJ02	Flashlight fish	
Protein coding genes, no.	2,447	3,290	873	4,442
Total TEs, no.	691	921	0	87
Transposase genes, no.	689	888	NA^a^	80
Other TEs (phage genes), no.	2	33	NA	7
Complete transposase genes, no.	0	0	NA	49
TE % of CDS	28	31	NA	2
Transposases by IS family, no.				
IS*5*	672	198		5
IS*982*		333		
IS*256*		75		
IS*L3*		57		
Tn*3*		25		
IS*200*/*605*		60		
IS*As1*				12
IS*66*		29		9
IS*6*	8			
Other and unknown	9	111		54

a NA, not applicable.

These results suggest an expansion of TEs in the anglerfish symbiont genomes. In order to better understand this pattern we categorized the identified transposase genes by insertion sequence (IS) family. In both symbiont genomes, transposase genes fell into a relatively small number of families (Table 4). Within the CC26 genome assembly, almost all transposases were members of IS family IS*5*, while in the MJ02 symbiont genome transposases were predominately from families IS*5*, IS*982*, and others. The transposase genes were relatively evenly spread across contigs within the well-assembled CC26 genome (see Fig. S7 in the supplemental material), with the exception of a few regions (>20 kb) with no insertions. These regions without transposons contained gene clusters or operons presumed to be necessary to the bacteria, including genes involved in cell division, cell wall synthesis, chemotaxis and motility, the tricarboxylic acid (TCA) cycle, and the luminescence (*lux*) gene operon. This pattern is consistent with large-scale expansions of a few transposons around the genomes, with some selection against insertion in necessary genes. The location of these transposons also suggests that their expansion may have facilitated gene loss in the genomes. All 55 pseudogenized functional genes (non-tranposase genes) within the MJ02 symbiont genome are adjacent to or interrupted by transposase fragments.

10.1128/mBio.01033-18.8 FIG S7  Chromosomal positions of transposase fragments in the CC26 symbiont genome. Gene content of large regions lacking TE insertions is indicated. Download FIG S7, DOCX file, 0.4 MB. Copyright © 2018 Hendry et al. 2018 Hendry et al. This content is distributed under the terms of the Creative Commons Attribution 4.0 International license.

**FIG 4:**
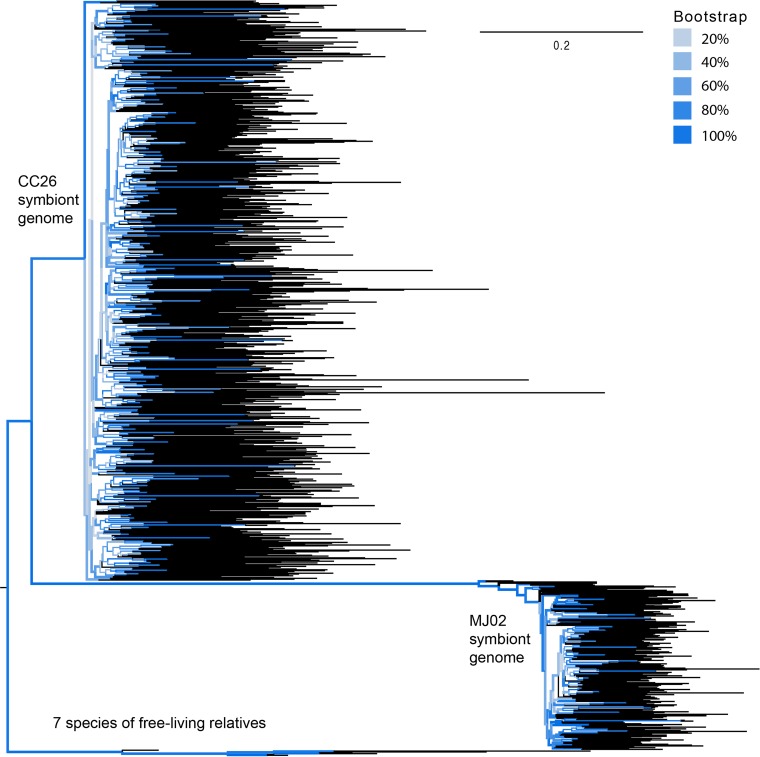
Phylogeny of IS*5* family transposases. Relationships among IS*5* family transposase genes and pseudogenes from the CC26 and MJ02 symbiont genomes, as well as 7 species of free-living relatives (Vibrio alginolyticus XSHD3, Vibrio anguillarum 775, Vibrio mimicus 6, Vibrio vulnificus YJ016, *Vibrio* sp. strain RC341, and Shewanella oneidensis MR-1) were reconstructed by maximum likelihood analysis. Bootstrap values are color coded, showing that the backbone of the tree has high bootstrap support. Tips are colored black.

All identified transposase genes in both anglerfish symbiont genomes were classified as pseudogenes, as they were all either missing the inverted-repeat regions typically found in the specific types of transposases or were truncated in aligned length compared to orthologs from close relatives. The very high number of transposase genes coupled with the fact that they are no longer functional suggests that each symbiont genome displays remnants of previous large-scale transposon proliferations. In order to investigate the relative timing of these events, we aligned transposases from each genome by family and performed phylogenetic analyses. IS*5* family transposases were expanded in both CC26 and MJ02 symbiont genomes, and IS*982* and IS*256* were expanded in just the MJ02 genome. IS*5* transposases from both genomes (Fig. 4) and IS*256* in the MJ02 genome (not shown) show a phylogenetic pattern consistent with a single expansion within each genome, with rapid diversification taking place over a short time scale, compared to typical rates of divergence of the same transposase groups in close relatives, and subsequent degeneration. IS*982* in the MJ02 genome shows a very similar pattern, although with possibly two expansions from distinct ancestral transposases within the genome (see Fig. S8 in the supplemental material). Such expansions could have occurred when a change in selective pressure on the bacterial genomes, such as the initiation of the symbiotic interaction between the bacteria and the host, allowed for a loss of regulation on transposable elements and therefore proliferation that was unchecked by purifying selection (24). This proliferation must have eventually been selected against after a large expansion, since all remaining transposases in the genome are nonfunctional. Both symbiont genomes appear to be at the tail end of a recent TE expansion, where constraint on remaining genes has halted continued transposase function, but the remnants of these genes remain in the bacterial genome.

10.1128/mBio.01033-18.9 FIG S8  Maximum likelihood tree of IS*982* family transposase fragments from the MJ02 symbiont genome, as well as functional IS*982* family transposase sequences from free-living relatives (Aliivibrio salmonicida LFI1238, Photorhabdus luminescens subsp. laumondii TTO1, Shewanella denitrificans OS217, and Shewanella oneidensis MR-1). Bootstrap values are color coded, showing that the backbone of the tree has high bootstrap support. Download FIG S8, DOCX file, 0.5 MB. Copyright © 2018 Hendry et al. 2018 Hendry et al. This content is distributed under the terms of the Creative Commons Attribution 4.0 International license.

Phylogenetic analysis of IS*5* family transposases found in both bacterial genomes suggests that the two anglerfish symbiont lineages may have independently undergone TE expansions. The IS*5* transposon expansion appears to have occurred at different time points within the “*Ca.* Enterovibrio luxaltus” and “*Ca.* Enterovibrio escacola” genomes rather than having occurred in the common ancestor of these symbionts. Compared to divergence between orthologs from different species of related bacteria, very long branches separate the IS*5* transposases from each symbiont (Fig. 4), with the expansion of IS*5* within “*Ca.* Enterovibrio escacola” possibly occurring more recently. Since anglerfish symbionts are not physically restricted inside host cells, genomic degeneration and TE expansions in the bacteria could have occurred separately from the initial establishment of the symbiosis, possibly at different times in each lineage. Alternatively, one or both of these symbiont lineages could be the result of a more recent symbiont replacement and subsequent genomic reduction.

### Conclusions.

A striking feature of the anglerfish symbiont genomes is the convergence in gene content and genomic patterns with flashlight fish symbionts. Like other luminous symbionts, these two groups of bacteria are maintained extracellularly yet have independently undergone a process of large-scale gene loss leading to likely host dependence. This raises the question of why other luminous symbionts have not evolved similarly and what features may be shared between these fish groups that could influence the evolution of their symbionts. One possibility is that opportunities for vertical transmission of symbionts could allow for genome reduction to begin. The known schooling behavior of flashlight fishes presents a possible mechanism for pseudovertical transmission of their symbionts through the environment to larvae developing near adults (5, 7, 17, 63). Ceratioid fish are nonschooling and lay buoyant eggs that hatch near the ocean surface (8). Ceratioid larvae do not develop near adults but have an ontogenic vertical migration in which the light organ gradually develops (8, 10). In this system, vertically transmitted bacteria would need to be transferred to eggs by adults and persist until the larval light organ is developed enough to be colonized or juveniles would need to encounter a colonized adult in the deep sea. Alternatively, ceratioids may acquire their symbionts from environmental populations, as do other deep-sea fish species (11, 64–66). It is not clear from the genome sequences of the symbionts which of these scenarios is more likely, but bacterial operational taxonomic units (OTUs [i.e., OTUs based on the V4 variable region of the 16S rRNA]) matching both “*Ca.* Enterovibrio luxaltus” and “*Ca.* Enterovibrio escacola” have been found in water samples taken from the same locations sampled here (77), consistent with environmental persistence and possible environmental acquisition of symbionts.

The extremely high number of transposase remnants in both anglerfish symbiont genomes demonstrates that the process of genome reduction is still ongoing in these lineages. Furthermore, the pattern of transposon expansions in each genome suggests these proliferations occurred at different times in each lineage, either because genomic reduction and TE expansions occurred in anglerfish symbiont lineages at different times rather than occurring coincidentally with bacteria becoming host associated, as is seen in intracellular symbionts, or because symbiont replacements have occurred in one or both lineages. These results highlight the importance of studying diverse symbiotic systems in order to better understand which patterns may be shared across systems and when symbioses may break with expectations.

## MATERIALS AND METHODS

Anglerfish specimens were collected during DEEPEND cruises in the Gulf of Mexico. Specimens were identified after collection by Tracey Sutton, and lures were immediately removed with a sterile scalpel and placed in ethanol. Specimens were stored at −80°C until processed by the Microbiology and Genetics Laboratory at Nova Southeastern University’s Halmos College of Natural Sciences and Oceanography. All microbial DNA isolations were conducted following the Earth Microbiome Project protocol with the Mo Bio PowerLyzer PowerSoil kit. Illumina sequencing libraries were made from samples CC32 and MJ02 using a NexteraXT kit, and a library for CC26 was constructed using a Swift Biosciences PCR-free kit. Paired-end libraries were sequenced with a 250-bp read length on an Illumina HiSeq2500 instrument at the Cornell University Institute of Biotechnology Biotechnology Resource Center Genomics Facility. Genome assembly was done using the Discovar *de novo* assembler. Contigs were then binned by tetranucleotide frequency and coverage depth in MetaBAT (67). The binned genomic contigs for CC26 and MJ02 were then annotated in RAST (68). All coding sequences predicted by RAST were then compared to the most recent UniRef90 database release (March 2017) (69). Coding sequences for which the RAST annotation differed from the UniRef best hit were manually checked. Loci were considered possible pseudogenes if they were <60% of the length of the best UniRef hit or showed <30% amino acid similarity. All possible pseudogenes were checked manually by BLAST in UniRef90. Phylogenetic trees were constructed using Bayesian or maximum likelihood methods with conserved housekeeping genes (20, 70, 71) or genome-wide conserved protein sequences (72). Tests of evolutionary rate were performed in PAML (73). Transposase sequences were identified using the ISfinder database (74). Accession numbers for all sequences used in this study (see Table S1) and comparison genomes (see Text S1) are available in the supplemental material.

10.1128/mBio.01033-18.1 TEXT S1  Supplemental materials and methods. Download TEXT S1, DOCX file, 0.2 MB. Copyright © 2018 Hendry et al. 2018 Hendry et al. This content is distributed under the terms of the Creative Commons Attribution 4.0 International license.

### Accession numbers.

Annotated genomes were submitted to GenBank under GenBank accession no. CP020660, CP020661, CP020662, CP020663, and NBYY00000000. Detailed methods are available in the supplemental material (Text S1).

### Data availability.

Data are publicly available through the Gulf of Mexico Research Initiative Information and Data Cooperative (GRIIDC) at https://data.gulfresearchinitiative.org (doi:10.7266/N70P0X3T).
