# Supplementary material for: Ongoing Transposon-Mediated Genome Reduction in the Luminous Bacterial Symbionts of Deep-Sea Ceratioid Anglerfishes
Source: mBio. 2018 Jun 26;9(3):e01033-18. doi: 10.1128/mBio.01033-18 (PMC6020299; doi:10.1128/mBio.01033-18)
Supplement: FIG S1 [file mbo003183948sf1.docx]

**Fig. S1.** Frequency spectrum of alternate bases present in symbiont genome reads from the (A) CC32 library and (B) MJ02 library. The majority of alternate bases in each library are at very low frequency (< 0.05%), suggesting that they are errors rather than intra-light organ variation of the symbiont population.
