# Supplementary material for: Ongoing Transposon-Mediated Genome Reduction in the Luminous Bacterial Symbionts of Deep-Sea Ceratioid Anglerfishes
Source: mBio. 2018 Jun 26;9(3):e01033-18. doi: 10.1128/mBio.01033-18 (PMC6020299; doi:10.1128/mBio.01033-18)
Supplement: FIG S2 [file mbo003183948sf2.docx]

**Fig. S2.** Maximum likelihood phylogenetic tree using 7 housekeeping genes (16S rRNA gene, *atpA*, *gapA*, *gyrB*, *pyrH*, *rpoA*, and *topA*). Analysis was done in IQTree using a general time reversible model (chosen by IQTree) and 1000 bootstrap replicates. Bootstrap values >80% are shown below nodes.
