# Supplementary material for: Ongoing Transposon-Mediated Genome Reduction in the Luminous Bacterial Symbionts of Deep-Sea Ceratioid Anglerfishes
Source: mBio. 2018 Jun 26;9(3):e01033-18. doi: 10.1128/mBio.01033-18 (PMC6020299; doi:10.1128/mBio.01033-18)
Supplement: FIG S3 [file mbo003183948sf3.docx]

**Fig. S3.** Maximum likelihood phylogenomic tree compared to a maximum likelihood phylogeny of *lux* luminescence genes. Phylogenomic analysis was done using PhyloPhlAn using the strains in Table S1, and *lux* gene analysis was done in IQTree using a general time reversible model (chosen by IQTree) and 1000 bootstrap replicates. Branches with > 80% bootstrap support are show in bold.
