# Supplementary material for: Ongoing Transposon-Mediated Genome Reduction in the Luminous Bacterial Symbionts of Deep-Sea Ceratioid Anglerfishes
Source: mBio. 2018 Jun 26;9(3):e01033-18. doi: 10.1128/mBio.01033-18 (PMC6020299; doi:10.1128/mBio.01033-18)
Supplement: FIG S4 [file mbo003183948sf4.docx]

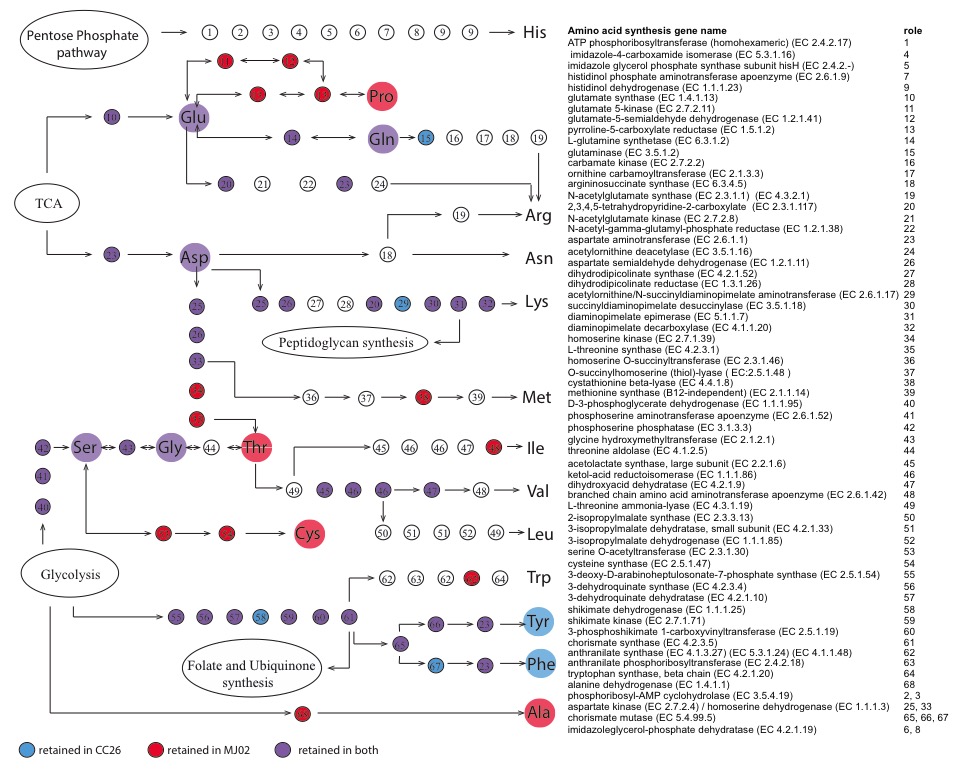


**Fig. S4.** Loss of amino acid synthesis pathways in anglerfish symbionts. Amino acid synthesis pathways present in relatives (taken from KEGG pathways from *Vibrio campbellii* ATCC BAA-1116) are shown with gene presence or absence in anglerfish symbiont genomes color-coded. Complete pathways are indicated by colored amino acid products. Numbered circles represent enzymatic steps in the pathway and their corresponding enzymes are listed on the right.
