# Supplementary material for: Ongoing Transposon-Mediated Genome Reduction in the Luminous Bacterial Symbionts of Deep-Sea Ceratioid Anglerfishes
Source: mBio. 2018 Jun 26;9(3):e01033-18. doi: 10.1128/mBio.01033-18 (PMC6020299; doi:10.1128/mBio.01033-18)
Supplement: FIG S5 [file mbo003183948sf5.docx]

**Fig. S5.** Numbers of genes in functional categories for free-living Vibrionaceae members, anglerfish symbionts, and flashlight fish symbionts. Genes may be present in multiple categories.
