# Supplementary material for: Ongoing Transposon-Mediated Genome Reduction in the Luminous Bacterial Symbionts of Deep-Sea Ceratioid Anglerfishes
Source: mBio. 2018 Jun 26;9(3):e01033-18. doi: 10.1128/mBio.01033-18 (PMC6020299; doi:10.1128/mBio.01033-18)
Supplement: FIG S6 [file mbo003183948sf6.docx]

**Fig. S6.** Maximum likelihood phylogenetic tree of methyl-accepting chemotaxis protein (MCP) sequences from anglerfish symbionts, flashlight fish symbionts, and free-living members of Vibrionaceae. Analysis was done in IQTree using a general matrix of amino acid exchange rates, empirically determined amino acid frequencies, a gamma distribution with four categories for rate heterogeneity and 1000 bootstrap replicates. Branches are color coded by bootstrap value. MCP genes previously demonstrated to contain conserve amino acid ligand binding domains (T. A. Hendry, J. R. de Wet, K. E. Dougan, P. V. Dunlap, Genome Biol Evol 8: 2203–2213, 2016, doi.org/10.1093/gbe/evw161) are highlighted. Sequences were taken from GenBank, with the addition of anglerfish symbiotn MPC proteins from this study.
