# Supplementary material for: Ongoing Transposon-Mediated Genome Reduction in the Luminous Bacterial Symbionts of Deep-Sea Ceratioid Anglerfishes
Source: mBio. 2018 Jun 26;9(3):e01033-18. doi: 10.1128/mBio.01033-18 (PMC6020299; doi:10.1128/mBio.01033-18)
Supplement: FIG S7 [file mbo003183948sf7.docx]

**Fig. S7.** Chromosomal positions of transposase fragments in the CC26 symbiont genome. Gene content of large regions lacking TE insertions is indicated.
