# Supplementary material for: Ongoing Transposon-Mediated Genome Reduction in the Luminous Bacterial Symbionts of Deep-Sea Ceratioid Anglerfishes
Source: mBio. 2018 Jun 26;9(3):e01033-18. doi: 10.1128/mBio.01033-18 (PMC6020299; doi:10.1128/mBio.01033-18)
Supplement: FIG S8 [file mbo003183948sf8.docx]

**Fig. S8.** Maximum likelihood tree of IS982 family transposase fragments from the MJ02 symbiont genome, as well as functional IS982 family transposase sequences from free-living relatives (*Aliivibrio salmonicida* LFI1238, *Photorhabdus luminescens* subsp. *laumondii* TTO1, *Shewanella denitrificans* OS217, and *Shewanella oneidensis* MR-1). Bootstrap values are color coded, showing that the backbone of the tree has high bootstrap support.
