# Supplementary material for: Ongoing Transposon-Mediated Genome Reduction in the Luminous Bacterial Symbionts of Deep-Sea Ceratioid Anglerfishes
Source: mBio. 2018 Jun 26;9(3):e01033-18. doi: 10.1128/mBio.01033-18 (PMC6020299; doi:10.1128/mBio.01033-18)
Supplement: TEXT S1 [file mbo003183948s1.docx]

**Text S1. Supplementary materials and methods.**

**Genome sequencing, assembly, and annotation.** Anglerfish specimens were collected during DEEPEND cruises aboard the R/V *Point Sur.* A six-net MOCNESS (Multiple Opening and Closing Net and Environmental Sensing System) (1) with 3-mm mesh was used for sample collection with 5 nets (N1-N5) set to sample at discrete depths and one net (N0) collecting samples from the surface to a max depth of approximately 1500 m. Sampling stations were selected based on the existing Southeast Area Monitoring and Assessment Program (SEAMAP) sampling grid (2). Specimen MJ02 was collected during the DP01 cruise at station B001 (TrawlStartLat,Long: 28.918,-87.998; TrawlEndLat,Long: 29.058,-88.02) on May 2, 2015. It was captured by Net0, which sampled from the surface (0 m) to a depth of 1143 m. CC26 and CC32 were collected during the DP02 cruise with CC26 collected at station B080 (TrawlStartLat,Long: 28.54901,-87.02955; TrawlEndLat,Long: 28.42853, 86.9372) on Aug 16, 2015 and CC32 collected at station SE3 (TrawlStartLat,Long: 26.93223,-87.02041; TrawlEndLat,Long: 27.01393, 86.98386) on August 19, 2015. CC26 was captured by Net0, which sampled from the surface to a depth of 751 m, while CC32 was captured by Net4, which sampled from 198 m to 597 m. Once onboard, specimens were sorted, identified, and lures were removed with a sterile scalpel and placed in ethanol or RNALater by Tracey Sutton. Specimens were stored at -80C until processed by the Microbiology & Genetics Laboratory at Nova Southeastern University’s Halmos College of Natural Sciences and Oceanography. All microbial DNA isolations were conducted following the Earth Microbiome Project protocol with the MO BIO PowerLyzer PowerSoil kit. Illumina sequencing libraries were made from samples CC32 and MJ02 using a NexteraXT kit and a library for CC26 was constructed using a Swift Biosciences PCR-free kit. Paired-end libraries were sequenced with a 250 bp read length on an Illumina HiSeq2500 instrument at the Cornell University Institute of Biotechnology Biotechnology Resource Center Genomics Facility.

Genome assembly was done using the Discovar *de novo* assembler. Contigs were then binned by tetranucleotide frequency and coverage depth in MetaBAT (3). For each *C. couesii* sample MetaBAT recovered one bin containing the longest contigs in the assembly as well as one or two fully circular plasmid contigs. Contigs from these bins showed high BLAST similarity to members of Vibrionaceae and distinct coverage depth averages compared to other bins containing contigs greater than 4 kb in length. Reciprocal BLAST between these bins from each *C. couesii* sample found that all contig sequences from each bin was represented in the other, with the exception of a circular plasmid from CC26 that was missing from the CC32 assembly. Comparisons with BLAST showed that the CC26 and CC32 genomes were 99.9% identical at the nucleotide level and therefore subsequent analyses focused on the better assembled CC26 symbiont genome. The symbiont bin from the CC26 library contained only two non-plasmid contigs which show similarity to chromosomes one and two from other Vibrionaceae taxa. The bin from the *M. johsonii* assembly that contained the longest contigs also contained a number of short contigs. All contigs in this bin were subjected to BLAST in GenBank and retained as part of the metagenome if they were larger than 5 kb and had high BLAST similarity to Vibrionaceae plasmids (with any coverage depth) or had high BLAST similarity to Vibrionaceae genomes and a similar coverage depth to the largest symbiont contigs. Fifty contigs falling outside of these requirements, 37 less than 5 kb in length and 13 ranging in size from 5 – 16 kb but with either very low (<0.5x) or high (>83x) coverage compared to the larger contigs (~58x) were removed from the metagenome. We note that we did not identify any apparent genes related to core metabolic functions within these discarded contigs.

We used CheckM (4) and the conserved proteins published in Raes et al. (5) to check the completeness of the binned genomes. Both methods recovered a high number of conserved markers in the genomes, with CheckM uncovering 293/312 markers for the CC26 symbiont genome and 288/312 markers for the MJ02 symbiont genome (estimated completeness of 91% and 90%, respectively) and 32/35 of the Raes et al. markers recovered. In both cases either all (Raes et al. method) or most (CheckM) markers not found were missing from both symbiont genomes, suggesting that they have been lost in the reduced genomes rather than being missing from the genome assembly. The actual completeness of the symbiont genomes is therefore likely higher than 90%.

To investigate possible genetic variation within each light organ symbiont population, we used Bowtie (6) to align library reads to the assembled reference symbiont genome. The MPileup function in SAMtools (7) was then used to find all alternate bases and their read depth. Only sites with at least 30x read depth were considered and alternate bases were binned by fraction of the read depth to determine their frequency. These results were consistent with patterns in other known bioluminescent symbioses wherein light organs are colonized by small numbers of strains and contain very little diversity (8, 9). Therefore, for ease and accuracy we subsequently refer to the binned symbiont contigs as genomes rather than metagenomes.

The binned genomic contigs for CC26 and MJ03 were then annotated in RAST (10). All coding sequences predicted by RAST were then compared to the most recent UniRef90 database release (March, 2017) (11). Coding sequences for which the RAST annotation differed from the UniRef best hit were manually checked and if these did not agree the gene was classified as unknown function. Loci were considered possible pseudogenes if they were <60% of the length of the best UniRef hit, or <30% amino acid similarity. All possible pseudogenes were checked manually by BLAST in UniRef90.

**Evolutionary relationships and rates.** Phylogenetic trees were constructed using both multiple housekeeping gene loci and genome wide conserved protein sequences. GenBank accession numbers and strain numbers for all sequences included in analysis are available in supplementary table S1. For housekeeping gene analyses seven loci (16S rRNA gene, atpA, gapA, gyrB, pyrH, rpoA, and topA), which are widely used for phylogenetic analysis in Vibrionaceae, were taken from the CC26, CC32 and MJ02 anglerfish symbiont assemblies, as well as from GenBank for 37 taxa in the family Vibrionaceae and two outgroup taxa (Table S1). Alignments were constructed in Clustal (12) and manually checked for possible errors. Maximum likelihood reconstruction was done using IQ-Tree (13) whereas a Bayesian phylogeny was made in Mr.Bayes (14). Both used a general time reversible model with a gamma distribution of rate heterogeneity and invariant sites. PhyloPhlAn (15) was used to extract 253 conserved protein sequences from the translated coding loci of 31 taxa in the family Vibrionaceae, the CC26 and MJ02 genome assemblies, and two outgroup taxa (Table S1). These proteins were aligned in Clustal and used for phylogenetic analysis in IQTree with a general matrix of amino acid exchange rates, empirically determined amino acid frequencies and a gamma distribution with four categories for rate heterogeneity.

Both the maximum likelihood housekeeping gene tree and phylogenomic protein tree were used in PAML (16) to determine if anglerfish symbionts are evolving at a faster rate than relatives. The codeml algorithm was used with either a global clock (equal rate null hypothesis) or a local clock with different rates for anglerfish symbionts and other clades. For comparison, obligately dependent flashlight fish symbionts were also constrained by a local clock. Model parameters previously determined by IQTree were used in each analysis. Models were retained if log likelihood ratio tests indicated significant improvement over the null hypothesis. Specific genes of interest, such as *lux* luminescence genes and MCP genes, were extracted from genomes with BLAST using orthologs from GenBank and checked against the annotation for confirmation. For phylogenetic reconstruction using these genes, MAFFT (17) was used to produce alignments and IQTree was used to phylogenies. BLAST was also used to calculate nucleotide similarity of coding regions between the CC26 and MJ02 genomes and ANI was calculated with a minimum similarity of 70% and a minimum alignment length of 200 bases between orthologous loci.

**Genomic comparisons.** For comparisons of gene content, genomes of the following Vibrionaceae strains were obtained from GenBank: *Aliivibrio fischeri* ES114 (GCA_000011805.1), *Aliivibrio wodanis* AWOD1 (GCA_000953695.1), *E. calviensis* DSM 14347 (GCF_000621165.1), *Enterovibrio coralii* CAIM 912 (GCA_001559595.1), *E. norvegicus* FF-33 (GCA_000286835.2), *Grimontia hollisae* CIP 101886 (GCA_000176515.1), *Grimontia indica* AK16 (GCA_000333895.2), *Photobacterium damselae* CIP 102761 (GCA_000176795.1), *Photobacterium mandapamensis* svers.1.1 (GCA_000211495.1), *Photobacterium profundum* SS9 (GCA_000196255.1), *Candidatus* “Photodesmus blepharus” Ppalp1 (GCA_000731795.1), *Candidatus* “Photodesmus katoptron” Akat8 (GCA_000731785.1), *S. costicola* subsp. *costicola* ATCC 33508 (GCA_000390145.1), *Vibrio campbellii* ATCC BAA-1116 (GCA_000390145.1), *Vibrio mimicus* MB451 (GCA_000390145.1), *Vibrio orientalis* CIP 102891 (GCA_000176235.1), and *Vibrio vulnificus* CMCP6 (GCA_000039765.1). These genome sequences were annotated as described above for the anglerfish symbiont genomes, in order to avoid variation caused by annotation methods. Metabolic pathways were checked for completeness using the *A. fischeri* ES114 pathways from the KEGG PATHWAY database (18).

**Transposon analysis.** Genes annotated in the anglerfish symbiont genomes as transposases by RAST were identified as hypothetical transposable elements. These transposases were compared by BLAST against the ISfinder database (19) to assign them to families and identify orthologs from close relatives. Orthologs from relatives were then used to search the symbiont genomes for incomplete transposase fragments. The CC26 symbiont genome was dominated by IS5 family transposases and was searched by BLAST using an IS5 transposase from a *Vibrio vulnificus* genome (ISVvu5 in ISfinder). The MJ02 symbiont had more diverse transposases present and was searched using the IS5 ortholog as well as an IS256 ortholog (ISVa19 from *Vibrio anguillarium*) and an IS982 ortholog (ISPlu6 from *Photorhabdus luminescens*). The symbiont genomes were searched by tblastn and when multiple hits overlapped in the same region of a contig only the best match hit was retained. Hits were retained as transposases if they matched with >30% amino acid sequence identity and >20% length to the query. Transposases were considered full length and functional if they matched by >60% amino acid similarity to the ortholog and they retained inverted repeat sequences. Extracted nucleotide transposase sequences from the same family were aligned by MAFFT. Trees were constructed for IS5 and IS982 family transposase genes in IQTree using a transversion model and gamma distribution of four rate categories whereas the IS256 family transposase tree used a general time reversible model.

**REFERENCES**

1. Wiebe PH, Burk KH, Boyd SH, Morton AW. 1976. A multiple openingclosing net and environmental sensing system for sampling zooplankton. J Mar Res 34:313–326.

2. Eldridge PJ. 1988. The southeast area monitoring and assessment program (SEAMAP): a state federal-university program for collection, management, and dissemination of fishery independent data and information in the southeastern United States. Mar Fish Rev 50:29–39.

3. Kang DD, Froula J, Egan R, Wang Z. 2015. MetaBAT, an efficient tool for accurately reconstructing single genomes from complex microbial communities. PeerJ 3:e1165.

4. Parks DH, Imelfort M, Skennerton CT, Hugenholtz P, Tyson GW. 2015. CheckM: assessing the quality of microbial genomes recovered from isolates, single cells, and metagenomes. Genome Res 25:1043–1055.

5. Raes J, Korbel JO, Lercher MJ, von Mering C, Bork P. 2007. Prediction of effective genome size in metagenomic samples. Genome Biol 8:R10.

6. Langmead B. 2010. Aligning short sequencing reads with Bowtie. Curr Protoc Bioinforma Ed Board Andreas Baxevanis Al CHAPTER:Unit-11.7.

7. Li H, Handsaker B, Wysoker A, Fennell T, Ruan J, Homer N, Marth G, Abecasis G, Durbin R, 1000 Genome Project Data Processing Subgroup. 2009. The Sequence Alignment/Map format and SAMtools. Bioinforma Oxf Engl 25:2078–2079.

8. Wollenberg MS, Ruby EG. 2009. Population structure of Vibrio fischeri within the light organs of Euprymna scolopes squid from Two Oahu (Hawaii) populations. Appl Environ Microbiol 75:193–202.

9. Hendry TA, Dunlap PV. 2014. Phylogenetic divergence between the obligate luminous symbionts of flashlight fishes demonstrates specificity of bacteria to host genera. Environ Microbiol Rep 6:331–338.

10. Overbeek R, Olson R, Pusch GD, Olsen GJ, Davis JJ, Disz T, Edwards RA, Gerdes S, Parrello B, Shukla M, Vonstein V, Wattam AR, Xia F, Stevens R. 2014. The SEED and the Rapid Annotation of microbial genomes using Subsystems Technology (RAST). Nucleic Acids Res 42:D206–D214.

11. Suzek BE, Wang Y, Huang H, McGarvey PB, Wu CH. 2015. UniRef clusters: a comprehensive and scalable alternative for improving sequence similarity searches. Bioinformatics 31:926–932.

12. Larkin MA, Blackshields G, Brown NP, Chenna R, McGettigan PA, McWilliam H, Valentin F, Wallace IM, Wilm A, Lopez R, Thompson JD, Gibson TJ, Higgins DG. 2007. Clustal W and Clustal X version 2.0. Bioinforma Oxf Engl 23:2947–2948.

13. Nguyen L-T, Schmidt HA, von Haeseler A, Minh BQ. 2015. IQ-TREE: A Fast and Effective Stochastic Algorithm for Estimating Maximum-Likelihood Phylogenies. Mol Biol Evol 32:268–274.

14. Ronquist F, Teslenko M, van der Mark P, Ayres DL, Darling A, Höhna S, Larget B, Liu L, Suchard MA, Huelsenbeck JP. 2012. MrBayes 3.2: Efficient Bayesian Phylogenetic Inference and Model Choice Across a Large Model Space. Syst Biol 61:539–542.

15. Segata N, Börnigen D, Morgan XC, Huttenhower C. 2013. PhyloPhlAn is a new method for improved phylogenetic and taxonomic placement of microbes. Nat Commun 4:2304.

16. Yang Z. 2007. PAML 4: phylogenetic analysis by maximum likelihood. Mol Biol Evol 24:1586–1591.

17. Katoh K, Standley DM. 2013. MAFFT Multiple Sequence Alignment Software Version 7: Improvements in Performance and Usability. Mol Biol Evol 30:772–780.

18. Kanehisa M, Goto S. 2000. KEGG: kyoto encyclopedia of genes and genomes. Nucleic Acids Res 28:27–30.

19. Siguier P, Perochon J, Lestrade L, Mahillon J, Chandler M. 2006. ISfinder: the reference centre for bacterial insertion sequences. Nucleic Acids Res 34:D32–D36.
